# Tropical Medicine

**Published:** 1908-09-05

**Authors:** 


### THE STUDY OF TROPICAL MEDICINE.

The recent advances in our knowledge of tropical diseases have rendered it imperative that special teaching by experts should be given to young graduates about to take up their different spheres of work in the various colonies. Thus have arisen, in most of the countries that possess tropical dependencies, special schools of training usually designated Schools of Tropical Medicine. Let us define what tropical medicine really means, and how it differs clinically and practically from the general medicine taught in the ordinary medical schools. As regards the first, the clinical aspect, there is not much to say; the methods employed are the same, but in the tropics, besides almost all the ordinary diseases of temperate climates, there are some peculiar forms, which are difficult to see and study at home.

As regards the second, the practical aspect of tropical medicine, we find much the same applies; the methods are again similar, the blood has to be examined in just the same way, bacteria have to be grown and studied, and so on. Where the difference comes in is that certain tropical diseases have as their causative agents peculiar parasites, often of a protozoal nature. Further, in addition to an elementary knowledge of protozoology, helminthology and entomology must be mastered by the would-be tropical expert, and this alone at once differentiates the scientific medicine of temperate climates from that of the hotter regions. This blending of the study of pure zoology with that of medicine is the most striking feature that has occurred in medicine within recent years. It has resulted in the production of voluminous works on mosquitoes and other insects previously hardly known or mentioned, and it has thrown fresh light and interest on the complicated study of the protozoa, raising that subject to a height of interest almost exceeding that of bacteriology. This of itself is sufficient to indicate a necessity of special instruction, and it places the practical side of tropical medicine at least on a plane with special subjects such as bacteriology, in which of course, post-graduate teaching is often required.

There are several classes of practitioners to whom a knowledge of tropical medicine is valuable. So many invalids are constantly coming from our colonial possessions to London that it is almost essential for the ordinary consultant, in the first place, to know something about tropical medicine. Secondly, graduates who intend to practise abroad, and who are leaving England for the first time, require the special instruction given at schools of tropical medicine before they set out for abroad.

The Navy, the Army, the Foreign Office, and the Colonial Office all insist now that their medical officers shall have instruction in tropical diseases before they take up their appointments, and this clearly shows the value they set on such instruction. The Army and Navy have their own schools of instruction, Colonial medical men are sent to the schools of tropical medicine at London or Liverpool, and these also afford facilities to private students, missionaries, and others. Most railway and mining companies in hot countries now give the preference to a medical man who can produce a certificate of a course of instruction from one or the other of the schools.

### SCHOOLS OF TROPICAL MEDICINE.

### The London School of Tropical Medicine.

It may interest intending applicants for colonial or other posts in the tropics if one gives a short *résumé* of what the course of instruction at the London School of Tropical Medicine consists of, and how long it lasts. The sessions extend over three months each, and there are three a year; the Colonial Office requires that men selected for posts in the tropics should attend one such session, and, in addition to showing regularity in attendance, should pass the examination at the end of the course. Failure to do so involves loss of the appointment. Other students may take the examination or not, as they choose, and, if successful, they are entitled to a certificate to that effect. The courses qualify, and are useful preparations, for the Diploma in Tropical Medicine and Hygiene granted by the University of Cambridge. The student is required to attend from 10 to 4 or 5 daily during the three months. The instruction is divided into three headings: (1) the practical work in the laboratory, by far the most important and useful of the three; (2) clinical instruction in the wards and the demonstration of cases; and (3) systematic lectures. Under the first heading the student is trained in hæmatology, protozoology, bacteriology, entomology, helminthology, pathology, and morbid anatomy; the tuition is eminently practical, the student preparing, staining, and mounting his own specimens, so that when he completes his studies he has in possession a very complete set of specimens, which serve him as standards for the future. Under the second heading comes the clinical instruction in the wards: the paucity of cases is a serious drawback in this part of the teaching, though, of course, one case studied thoroughly is of much more value than many simply glanced at, but still at the same time this is a real defect, and one which it is difficult to remedy.

### The Liverpool School of Tropical Medicine.

This school is carried on in conjunction with the University of Liverpool and the Royal Southern Hospital, for the purposes of research and clinical teaching respectively. A special ward at the hospital has been set apart for the reception and treatment of tropical diseases, and facilities for investigation and study are given in the Johnston Laboratories of the University. The fee for a full course (lasting about two months) is 10 guineas. A hall of residence is attached to the school. A vast amount of research has been accomplished at this school in addition to the ordinary tropical courses for the younger graduate, and any medical man from the tropics who wishes a helping hand in this direction cannot do better than go there.

---

The University of Edinburgh grants a diploma in tropical medicine and hygiene. The examinations are held in January and July. The fees for the first and any subsequent appearance are: Practical bacteriology, £1 1s.; diseases of tropical climates, £1 1s.; tropical hygiene, £1 1s.; tropical clinical medicine, £1 1s.; total, £4 4s.

